# CaCO_3_-Assisted Preparation of pH-Responsive Immune-Modulating Nanoparticles for Augmented Chemo-Immunotherapy

**DOI:** 10.1007/s40820-020-00549-4

**Published:** 2020-11-22

**Authors:** Yujie Zhu, Zhijuan Yang, Ziliang Dong, Yimou Gong, Yu Hao, Longlong Tian, Xianzhu Yang, Zhuang Liu, Liangzhu Feng

**Affiliations:** 1 Jiangsu Key Laboratory for Carbon-Based Functional Materials and Devices, Institute of Functional Nano and Soft Materials (FUNSOM), Soochow University, Suzhou, 215123 Jiangsu People’s Republic of China; 2 Institutes for Life Sciences, School of Medicine, South China University of Technology, Guangzhou, 510006 Guangdong People’s Republic of China

**Keywords:** CaCO_3_-assisted double emulsion, pH-responsiveness, Neutralization of acidic TME, Immunosuppressive tumor microenvironment modulation, Chemo-immunotherapy

## Abstract

Due to the negative roles of tumor microenvironment (TME) in compromising therapeutic responses of various cancer therapies, it is expected that modulation of TME may be able to enhance the therapeutic responses during cancer treatment. Herein, we develop a concise strategy to prepare pH-responsive nanoparticles via the CaCO_3_-assisted double emulsion method, thereby enabling effective co-encapsulation of both doxorubicin (DOX), an immunogenic cell death (ICD) inducer, and alkylated NLG919 (aNLG919), an inhibitor of indoleamine 2,3-dioxygenase 1 (IDO1). The obtained DOX/aNLG919-loaded CaCO_3_ nanoparticles (DNCaNPs) are able to cause effective ICD of cancer cells and at the same time restrict the production of immunosuppressive kynurenine by inhibiting IDO1. Upon intravenous injection, such DNCaNPs show efficient tumor accumulation, improved tumor penetration of therapeutics and neutralization of acidic TME. As a result, those DNCaNPs can elicit effective anti-tumor immune responses featured in increased density of tumor-infiltrating CD8^+^ cytotoxic T cells as well as depletion of immunosuppressive regulatory T cells (Tregs), thus effectively suppressing the growth of subcutaneous CT26 and orthotopic 4T1 tumors on the Balb/c mice through combined chemotherapy & immunotherapy. This study presents a compendious strategy for construction of pH-responsive nanoparticles, endowing significantly enhanced chemo-immunotherapy of cancer by overcoming the immunosuppressive TME.

**Electronic supplementary material:**

The online version of this article (10.1007/s40820-020-00549-4) contains supplementary material, which is available to authorized users.

## Highlights

- CaCO_3_-assisted double emulsion method was developed to prepare pH-responsive nanoparticles enabling effective encapsulation of diverse molecules.
- The introducing of CaCO_3_ could enable deep intratumoral penetration of therapeutic agents and effective neutralization of acidic tumor pH to favor the reverse of immunosuppressive tumor microenvironment (TME).
- The reverse of immunosuppressive TME ascribing to NLG919-mediated IDO1 inhibition and CaCO_3_-mediated neutralization of acidic TME could collectively contribute to augmented chemo-immunotherapy of cancer.

**Electronic supplementary material:**

The online version of this article (10.1007/s40820-020-00549-4) contains supplementary material, which is available to authorized users.

## Graphical abstract

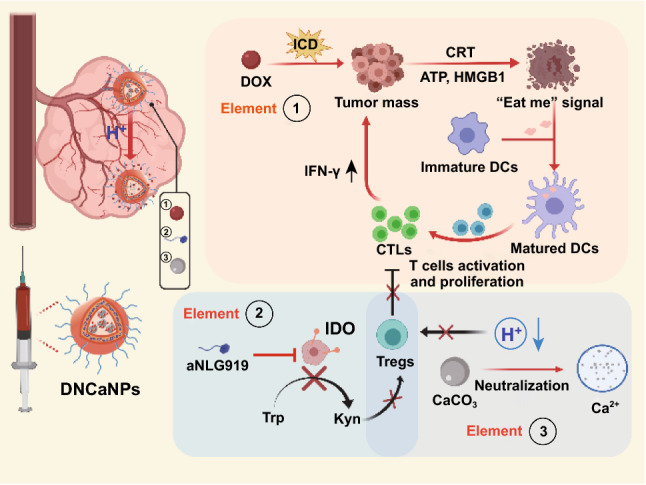

## Introduction

Emerging evidences have shown that the tumor microenvironment (TME) would foist a series of biological barriers to retard effective cancer treatment [1–5]. As a mainstream cancer treatment modality in the clinic, chemotherapy is far from satisfactory due to its limited therapeutic efficacy and severe side effects [6–9]. To achieve effective cancer chemotherapy with high safety, various nanoscale drug delivery systems enabling tumor-targeted delivery of chemotherapeutics have been intensively explored in the past decades [10–13]. Recent progresses have demonstrated that the clinically used nanodrugs, doxil and abraxane, exhibit significantly improved pharmacokinetic behaviors as well as greatly reduced side effects in comparison with their free drug counterparts [14]. However, these nanodrugs still show limited improvements in the therapeutic efficacy owing to the dense extracellular matrix and high interstitial fluid pressure inside the solid tumors, which negatively restrict the intratumoral diffusion of chemotherapeutics [15–18].

On the other hand, the immunosuppressive TME is another obstacle inside solid tumors that negatively impair the therapeutic efficacy of most chemotherapeutics by disabling the host’s immune system [19–21], even though certain types of chemotherapeutics could induce immunogenic cell death (ICD) to boost the immune attack of tumor cells [22–24]. It has been identified that overexpression of IDO1 is an immunosuppressive feature in a range of tumor types by catalytically converting essential tryptophan (Trp) to kynurenine (Kyn), which is characterized as an immunosuppressive molecule to induce the acquired peripheral tolerance by causing cell cycle arrest and death of effector T cells and in meanwhile activate the immunosuppressive regulatory T cells (Tregs) [25–27]. Recent studies have shown that tumor-targeted delivery of small molecule inhibitors toward the IDO1 could efficiently reverse the tumor immunosuppression and then enhance the therapeutic efficacy of chemotherapy and other cancer treatment modalities by activating the host’s immune system [28–30].

**Fig. 1:**
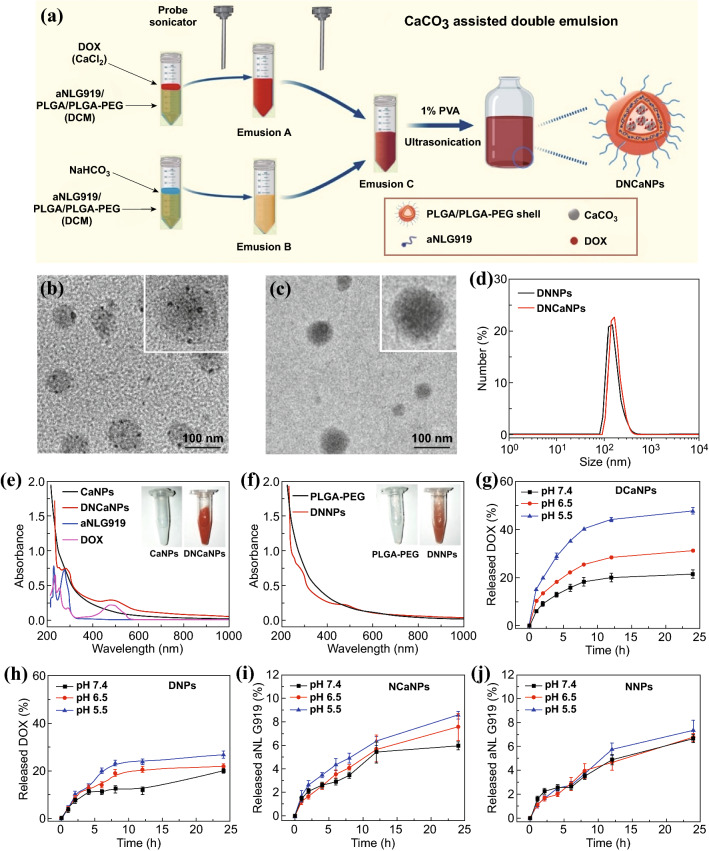
Preparation and characterization of the DNCaNPs. **a** A scheme illustrating the preparation process of such DNCaNPs via our proposed CaCO_3_-assisted double emulsion method. **b**, **c** Representative TEM images of DNCaNPs (**b**) and DNNPs (**c**). **d** DLS size distribution behaviors of DNNPs and DNCaNPs. **e, f** UV–Vis-NIR spectra of DNCaNPs (**e**) and non-responsive DNNPs without CaCO_3_ (**f**) and other formulation as indicated. Insets were the photographs of CaNPs, DNCaNPs, PLGA-PEG, and DNNPs as indicated. **g, h** DOX release profiles of DCaNPs (**g**) and DNPs (**h**) incubated at pH 7.4, 6.5, and 5.5. **i**, **j** aNLG919 release profiles of NCaNPs (**i**) and NNPs (**j**) incubated at pH 7.4, 6.5, and 5.5

To overcome the penetration barrier and immunosuppression of TME during treatment of solid tumors, it is meritorious to develop a suitable delivery system to allow co-delivery of ICD stimulus plus IDO1 inhibitors with deep intratumoral penetration to achieve synergistic effects of chemo-immunotherapy. In this study, we developed a concise strategy for the preparation of pH-responsive nanoparticles by introducing CaCO_3_ to the internal water phase during the classical double emulsion method to formulate poly(ethylene glycol)-b-poly(lactic-co-glycolic acid) (PLGA-PEG) nanoparticles (Fig. 1a). By employing the versatile molecular adsorption capacity of CaCO_3_, the obtained CaCO_3_@PLGA-PEG nanoparticles (CaNPs) showed significantly improved loading efficiencies to a range of distinct molecules compared to its counterpart PLGA-PEG nanoparticles prepared via the classical method. Then, both doxorubicin (DOX), a chemotherapeutic ICD inducer, and alkylated NLG919 (aNLG919), an inhibitor of IDO1, were simultaneously encapsulated via our CaCO_3_-assisted double emulsion method, obtaining DOX&aNLG919-coloaded CaNPs (DNCaNPs) with pH-responsive release profiles. It was found that such DNCaNPs after tumor accumulation could enable efficient intratumoral penetration of its therapeutic payloads, which were released when extravasated from the leaky blood vessels into the acidic tumor mass, in meanwhile neutralize the acidic TME by reacting with protons within solid tumors. Moreover, such DNCaNPs upon administration could not only directly cause effective ICD of cancer cells and then elicit the host’s antitumor immunity, but also reduce the density of immunosuppressive Tregs inside TMEs by synergistically inhibiting IDO1 and neutralizing tumor acidity. As a result, superior tumor growth suppression effects could be achieved by intravenous (i.v.) injection of DNCaNPs for both subcutaneous CT26 colon tumors and orthotopic 4T1 breast tumors. Consequently, this study presents a compendious CaCO_3_-assisted method for the preparation of pH-responsive nanomedicine with profound TME modulation effect for efficient chemo-immunotherapy of cancer.

## Experimental

### Materials

PEG_5k_-PLGA_10k_ was synthesized according to reported methods [31]. Dodecanoic acid was obtained from MACKLIN. NLG 919 was obtained from PharmaBlock Sciences (Nanjing), Inc. Doxorubicin hydrochloride (DOX) was obtained from Beijing HuaFeng United Technology Co. Ltd. Dichloromethane (CH_2_Cl_2_, DCM), sodium bicarbonate (NaHCO_3_), and CaCl_2_ were obtained from Sinopharm Chemical Reagent Co., Ltd., China. Poly (D,L-lactic-co-glycolic acid) (PLGA) and polyvinyl alcohol (PVA) were obtained from Sigma-Aldrich. Antibodies against cell surface markers for flow cytometry assay were purchased from eBioscience. All reagents (analytical grade) were used as received without further purification. Ultrapure water was obtained from a Milli-Q system (Health Force Bio-Meditech Holdings Ltd.).

### Synthesis of Alkylated NLG919

To synthesize alkylated NLG919, NLG919 (56 mg, 0.2 mmol) was dissolved in 5 mL DCM followed by being mixed with dodecanoic acid (60 mg, 0.3 mmol), 1-(3-dimethylaminopropyl)-3-ethyl carbon diimine hydrochloride (EDC, 76 mg, 0.4 mmol), 4-dimethylaminopyridine (DMAP, 6 mg, 0.05 mmol), N,N-diisopropyl ethylamine (DIPEA, 83 μL, 0.5 mmol) and stirred at room temperature for 24 h. Then, the product was collected via the vacuum rotary evaporation and re-dissolved with 20 mL DCM and successively washed with hydrochloric acid solution (0.01 M), saturated sodium NaHCO_3_ solution, and saturated NaCl solution before being dried with anhydrous sodium sulfate (Na_2_SO_4_). After that, such crude products were obtained by filtration and vacuum distillation and purified by using the column chromatography (eluent: N-hexane/ethyl acetate = 2/1). The obtained final products were confirmed by its ^1^H NMR spectrum and ^13^C NMR spectrum. ^1^H NMR (600 MHz, CDCl_3_) δ 7.71 (s, 1H), 7.54 (d, J = 7.8 Hz, 1H), 7.52 (d, J = 7.8 Hz, 1H), 7.35 (t, J = 7.8 Hz, 1H), 7.24 (d, J = 7.8 Hz, 1H), 7.17 (s, 1H), 5.09–5.07 (m, J = 1H), 5.06–5.04 (m, 1H), 2.39–2.34 (m, 1H), 2.27- 2.16 (m, 2H), 2.12–2.07 (m, 1H), 1.74–1.71 (m, 2H), 1.65–1.64 (m, 3H), 1.59–1.54 (m, 2H), 1.50–1.46 (m, 1H), 1.27–1.08 (m, 19H), 1.02–0.95 (m, 2H), 0.87 (t, J = 7.2 Hz, 3H). ^13^C NMR (151 MHz, CDCl3) δ 173.51, 144.52, 137.55, 131.38, 130.12, 128.66, 126.63, 124.18, 120.29, 118.62, 73.50, 57.79, 42.26, 37.95, 34.43, 32.02, 29.83, 29.71, 29.60, 29.44, 29.39, 29.30, 28.72, 28.11, 26.38, 26.07, 25.11, 22.81, 14.24.

### Preparation and Characterization of the DNCaNPs

DNCaP nanoparticles were prepared via the modified W1/O/W2 double emulsion method. In brief, to prepare emulsion A or B, 250 μL of NaHCO_3_ aqueous solution (0.625 M) or CaCl_2_ aqueous solution (1.25 M, with 1 mg DOX) was added to 750 μL of DCM containing PLGA (15 mg mL^−1^), PLGA-PEG (15 mg mL^−1^), and aNLG919 (4 mg mL^−1^), respectively, before being sonicated using an probe sonicator for 99 pulses (pulses on: 3 s, pulse off: 7 s). Then, emulsion A and emulsion B were mixed and sonicated using the probe sonicator for 99 pulses as aforementioned, obtaining emulsion C to be dropwisely added to 3 mL of PVA solution (1 wt%) under a water bath sonicator for 5 min and stirred at room temperature overnight to evaporate DCM. After that, the obtained DNCaNPs were washed three times by centrifugation at 14,800 rpm for 30 min to remove excess PVA and free drugs, followed by being centrifuged at 3000 rpm for 5 min to remove unwanted large particles. Other control nanoparticles used in this study were prepared via the same procedure apart from corresponding components that were not added.

The morphologies of the DNCaNPs were observed using a Tecnai F20 transmission electron microscope (TEM). The UV–Vis–NIR spectrum and dynamic light scattering (DLS) size distribution of DNCaNPs were measured by using an UV–Vis spectrometer (GENESYS™ 10S, Thermo) and a Malvern Zetasizer (Nano ZS90 Malvern), respectively. The amounts of DOX were quantified by recording their absorbance at 490 nm with a molar extinction coefficient of 10,500 mol^−1^ L cm^−1^ as our previously used methods [32]. The amounts of aNLG919 were quantified by recording their absorbance at 280 nm with a molar extinction coefficient of 17,431 mol^−1^ L cm^−1^.

### pH-Responsive DOX and aNLG919 Release Profile of DNCaNPs

To examine their pH-responsive DOX release profiles, DCaNPs (DOX = 0.5 mg) were transferred to dialysis tubes (molecular weight cutoff = 3500 Da), which were then immersed in 10 mL buffer solutions at pH 7.4, 6.5, and 5.5 solution and stirred at room temperature. Then, the absorbance of external solvents at 490 nm withdrawn at designated time points was recorded to determine the amounts of released DOX as aforementioned.

To examine the pH-responsive aNLG919 release profiles, NCaNPs (aNLG919 = 2.5 mg) were transferred to dialysis tubes (molecular weight cut-off = 3500 Da), which were then immersed in 10 mL buffer solutions at pH 7.4, 6.5, and 5.5 solution and stirred at room temperature. Then, the absorbance of external solvents withdrawn at designated time points at 280 nm was recorded to determine the amounts of released aNLG919 as aforementioned.

### In Vitro Cellular Uptake and Penetration of the DNCaNPs

CT26 murine colon cancer cells and 4T1 murine breast cancer cells were cultured under the standard conditions (37 °C and 5% CO_2_). For the preparation of CT26 MCSs, single-cell suspension of CT26 cells (1000 cells / well) was added into the 96-well plate, which was precoated with sterilized 1 wt% agar, and cultured at 37 °C for a week according to the previously reported method [33].

To evaluate the intracellular trafficking profiles of DNCaNPs, preseeded CT26 cells were incubated with DNCaNPs or non-responsive DNNPs at the same DOX concentration (10 μM) for 1, 2, 4, or 8 h before being washed twice with fresh phosphate-buffered saline (PBS), fixed with 4% paraformaldehyde, counterstained with DAPI (1 μg mL^−1^) for subsequent microscopic observation.

To exploit the pH-responsive penetration ability of DNCaNPs, CT26 MCSs at diameters of ~ 400 μm were incubated with DNCaNPs or DNNPs (DOX = 5 μM) at pH 7.4 and 6.5, respectively. These treated CT26 MCSs were collected after 12 h, washed twice with PBS, cryosectioned, and counterstained with DAPI before being subjected to a confocal microscopy (Zeiss LSM 800) as aforementioned.

### IDO-1 Inhibitory Capacity of DNCaNPs

To evaluate the IDO inhibitory activity of DNCaNPs, CT26 cells preseeded in a 96-well plate at a density of 5 × 10^3^ cells/well were simultaneously incubated with recombinant murine IFN-γ (50 ng mL^−1^) and various agents for 48 h. Then, the supernatant of each well (150 μL) was transferred to a new 96-well plate, followed by being added with 30% trichloroacetic acid (75 μL) and kept at 50 °C for another 6 h to ensure complete hydrolysis of formed N-formylkynurenine to kynurenine. After that, the supernatants were transferred to another new 96-well plate and incubated with an equal volume of Ehrlich reagent (2% p-dimethylamino-benzaldehyde in glacial acetic acid, w/v) for 10 min at room temperature before recording their absorbance at 490 nm by using a microplate reader.

To evaluate the ratio of Trp/Kyn in the cell supernatants after different treatments, CT26 cells preseeded in a 24-well plate at a density of 2 × 10^4^ cells/well were simultaneously incubated with recombinant murine IFN-γ (50 ng mL^−1^) and various agents for 48 h. Then, the supernatant of each well (300 μL) was transferred to a new 24-well plate, followed by being added with 30% trichloroacetic acid (150 μL) and kept at 50 °C for another 6 h to precipitate the proteins and ensure complete hydrolysis of formed N-formylkynurenine to kynurenine. After that, the concentrations of both Try and Kyn in the cell supernatants of these CT26 cells were quantitatively determined by high-performance liquid chromatography (HPLC, Thermo, UltiMate 3000).

### ICD Induction Capacity of DNCaNPs

To test the extracellular secretion of ATP, CT26 cells were seeded in the 24-well plate at a density of 2 × 10^4^ cells/well. After 12-h preincubation, CaNPs, NCaNPs, DcaNPs, and DNCaNPs at a DOX concentration of 5 μM were added and incubated for 24 h. Then, the supernatant of each well was collected with its ATP concentration determined using an ATP assay kit according to manufacturer’s instructions.

To evaluate CRT expression and HMGB1 release, CT26 cells preseeded in a 24-well plate containing 15-mm-circle glass cover were incubated with free DOX, DNCaNPs, and other indicated materials at an equivalent DOX concentration of 5 μM for 4 or 12 h, respectively. Then, those cells were washed with PBS, fixed with 4% paraformaldehyde, and stained with CRT or HMGB1 primary antibodies and corresponding fluorophore-conjugated secondary antibodies, respectively, according to the manufactures’ procedures.

### In Vitro Therapeutic Efficacy of DNCaNPs

To evaluate the cell killing ability of such DNCaNPs, CT26 cells preseeded in the 96-well plate at a density of 1 × 10^4^ cells/well were incubated with CaNPs, aNLG919, free DOX, DCaNPs, and DNCaNPs at a series concentration of DOX and corresponding concentrations of aNLG919. 48 h later, the relative cell viabilities after various treatments were determined by the standard methyl thiazolyl tetrazolium (MTT) assay.

Additionally, when their diameter approached to ~ 400 μm, these CT26 MCSs (*n* = 5) were incubated with DNCaNPs, DCaNPS, or DNNPs (DOX = 10 μM) at pH 7.4 and 6.5 for 4 days. The optical images of these treated CT26 MCSs were recorded every day, and their diameters were measured using the ImageAnalysis software (PerkinElmer).

### In Vivo Fluorescence Imaging and Pharmacokinetic Profiles of DNCaNPs

Female Balb/c mice were obtained from and used under the protocols approved by the Laboratory Animal Center of Soochow University. To establish subcutaneous CT26 tumor model, 2 × 10^6^ CT26 cells were subcutaneously injected into the right back of each mouse. To establish orthotopic 4T1 tumor model, 2 × 10^6^ 4T1 cells were injected into the fat pat of the left mammary gland of each mouse.

For in vivo fluorescence imaging, CT26 tumor-bearing mice were intravenously injected with DiR-labeled DNCaNPs and DNNPs and imaged under an in vivo imaging system (Lumina III IVIS, PerkinElmer) at designated time points. At 24 h p.i., these mice were killed with their main organs and tumors collected for ex vivo imaging.

To investigate the pharmacokinetics behavior of DNCaNPs, CT26 tumor-bearing mice *n* = 3) were i.v. injected with 200 μL of DNCaNPs and free DOX at a DOX dose of 5 mg kg^−1^. After that, blood samples (~ 20 μL) were collected from each mouse at indicated time points and lysed for the measurement of DOX fluorescence intensity under a Thermo Scientific Varioskan Flash microreader.

For intravital microscopic observation, CT26 tumor-bearing mice were anesthetized and an incision was made around the tumor without injuring the vessels and then attached with a cover slip [34–37]. Subsequently, these mice were i.v. injected with Cy5.5-loaded DNCaNPs or DNNPs and subjected to the confocal microscopy (Zeiss LSM 800) and imaged at designated time intervals.

### In Vivo Combination Therapy

For in vivo treatment, subcutaneous CT26 and orthotopic 4T1 tumor-bearing mice were randomly divided into five groups (*n* = 5) and treated by intravenous injection of saline (group I), CaNPs (group II), NCaNPs (group III), DCaNPs (group IV), and DNCaNPs (group V) at day 0, 3, and 6. The injection doses of DOX and aNLG919 for related groups were 5 and 25 mg kg^−1^, respectively. Since the beginning of treatment, the length (L) and width (W) of each tumor were recorded using a digital camera and the tumor volume was calculated by using the formula: *V* = *L* × *W* × *W*/2. The body weight of each mouse was recorded using a digital balance every other day throughout the whole treatment process. At 24 h after the treatments (day 7), one mouse from each group was killed and their tumor was harvested for TUNEL and H&E staining.

### Analysis of Anti-tumor Mechanism

To monitor the pH value within tumors, an invasive pH microelectrode (PM-HP5, PreSens) was inserted into the tumors on mice at 0 and 24h post-intravenous injection of CaNPs or PLGA-PEG nanoparticles, respectively (*n* = 3).

To evaluate the intratumoral CRT expression and HMGB1 release, 15 CT26 tumor-bearing mice were treated as aforementioned (*n* = 3). At 24 h after the treatments (day 7), these tumors after various treatments were collected, cryosectioned, and stained for the detection of intratumoral CRT expression and HMGB1 release via the immunofluorescence staining.

For the analysis of immune responses of these mice after various treatments, a total of 20 mice bearing CT26 tumor were treated as aforementioned (*n* = 4). At 1 day after the last injection (day 7), those mice were killed with lymph nodes close to the tumor and tumors collected, homogenized, and stained with varying antibodies by following our previously used protocols [38]. For the staining of DCs, these homogenized lymph node suspensions were sequentially stained with anti-CD11c-FITC, anti-CD80-PE, and anti-CD86-APC antibodies before being subjected to flow cytometric analysis. To stain effector T cells, the homogenized tumor mass suspensions were sequentially stained with anti-CD3-FITC, anti-CD4-APC, and anti-CD8-PE antibodies before being subjected to flow cytometric analysis. To stain Tregs, these homogenized tumor mass suspensions were sequentially stained with anti-CD3-FITC, anti-CD4-APC, anti-ForxP3-PE before subjected to flow cytometric analysis. The intratumoral secretion levels of IFN-γ were analyzed using a commercial ELISA kit according to the manufacturer’s protocols.

### Statistical Analysis

All the data were presented as mean ± SD. One-way analysis of variance (ANOVA) was used to determine the significance of the difference. Statistical significance was set at *p < 0.05, **p < 0.01, ***p < 0.001.

## Results and Discussion

### Preparation and Characterization of DNCaNPs

The classical water–oil–water (W1/O/W2) double emulsion method has been commonly utilized for the encapsulation of both hydrophilic and hydrophobic molecules, but its loading efficiency toward hydrophilic molecules may be limited [39]. Herein, by employing the strong adsorption of biocompatible CaCO_3_ to various types of hydrophilic molecules, we developed a novel strategy by introducing CaCO_3_ to the classical double emulsion process, termed as “CaCO_3_-assisted double emulsion method,” for efficient encapsulation of various hydrophilic molecules (Fig. 1a). After detailed optimization, CaCO_3_-containing nanoparticles (CaNPs) formulated with a mass feeding ratio of NaHCO_3_: CaCl_2_: PLGA: PLGA-PEG = 1: 2.6: 2.2: 2.2 were selected for further experiments. Notably, such CaNPs showed greatly improved encapsulation efficiencies toward hydrophilic payloads including DOX (9.1-fold), mitoxantrone (2.1-fold), chlorin e6 (9.8-fold), and bovine serum albumin (BSA, 2.9-fold), compared to those loaded in counterpart PLGA/PLGA-PEG (PLGA-PEG) nanoparticles obtained via the classical method without CaCO_3_ (Fig. S1). Therefore, the greatly improved encapsulation efficiencies of these hydrophilic molecules toward CaNPs should be ascribed to their strong coordination effects with CaCO_3_ according to previous studies [40].

In addition to loading of hydrophilic molecules, those CaNPs were further loaded with hydrophobic therapeutics. Commercial NLG919 molecules were conjugated with dodecanoic acid to increase its hydrophobicity (Fig. S2). The aNLG919 showed comparable inhibition effect on IDO1, but remarkably elevated encapsulation efficiency in comparison with commercial NLG919 (Figs. S3 and S4). Via simultaneous encapsulation of DOX and aNLG919 into the cavity and polymeric layer, respectively, the obtained DOX&aNLG919 co-loaded CaNPs (DNCaNPs) showed a mean size of 100 nm as visualized under the transmission electron microscopy (TEM) imaging (Fig. 1b). Moreover, we observed clearly dark dots inside such DNCaNPs when we were directly observed via the TEM imaging. These dark dots should be CaCO_3_ nanoparticles formed during the encapsulation process. In contrast, the TEM image of the PLGA/PLGA-PEG formula without CaCO_3_ (DNNPs) showed no dark dots inside (Fig. 1c). It was found that the content of CaCO_3_ in the obtained CaNPs was quantified to be 17.6% by using a commercial calcium colorimetric assay kit, and such CaNPs could rapidly react with proton to neutralize the acidic solutions as expected (Fig. S5). Via the dynamic light scattering (DLS) measurement, such DNCaNPs exhibited similar size distribution profile including mean diameters and polydispersity indexes to its counterpart DNNPs (Fig. 1d and Table S1), and consistent size distribution upon being incubated within H_2_O, phosphate-buffered saline (PBS), 10% fetal bovine serum (FBS) solution for up to 48 h (Fig. S6a), indicating them with excellent physiological stability. As measured under the UV–Vis spectrometer, the DNCaNPs showed characteristic absorption peaks of aNLG919 and DOX at 280 and 490 nm, respectively (Fig. 1e), indicating their successful encapsulation. The similar result was also observed in DNNPs formula (Fig. 1f).

Ascribing to the pH-responsive decomposition of CaCO_3_, such DCaNPs showed more efficient DOX release under acidic pHs, with about 49.3%, 31.0%, and 19.1% of DOX released upon incubation at pH 5.5, 6.5, and 7.4 for 24 h, respectively (Fig. 1g). In sharp contrast, DNPs prepared without introducing CaCO_3_ exhibited similar DOX release behaviors after being incubated in buffers with varying pH values (Fig. 1h). Additionally, relatively slow and continuous aNLG919 release profiles from both NCaNPs and DNPs were observed since aNLG919 was loaded in the hydrophobic PLGA shell, enabling the long-lasting regulation of immune microenvironment in tumor (Fig. 1i, j). Then, the time-dependent size distribution and morphology evolution behaviors of both DNCaNPs and DNNPs incubated at pH 5.5, 6.5, and 7.4 were carefully studied. It was shown that both DNCaNPs and DNNPs exhibited consistent size distribution profiles upon being incubated at varying pHs for up to 24 h as measured via the DLS measurement (Fig. S6b). Under the TEM imaging, obvious small cavities were observed inside such DNCaNPs upon being incubated at pH 5.5 and 6.5 for 24 h, but not observed inside those incubated at pH 7.4 (Fig. S6c). Such distinctive morphology should be ascribed to the acidic pH-induced decomposition of CaCO_3_ inside those DNCaNPs. Taken together, these results demonstrate that such DNCaNP is a promising pH-responsive drug delivery carrier.

### In Vitro pH-Responsive Cellular Uptake and Penetration Behaviors of DNCaNPs

**Fig. 2:**
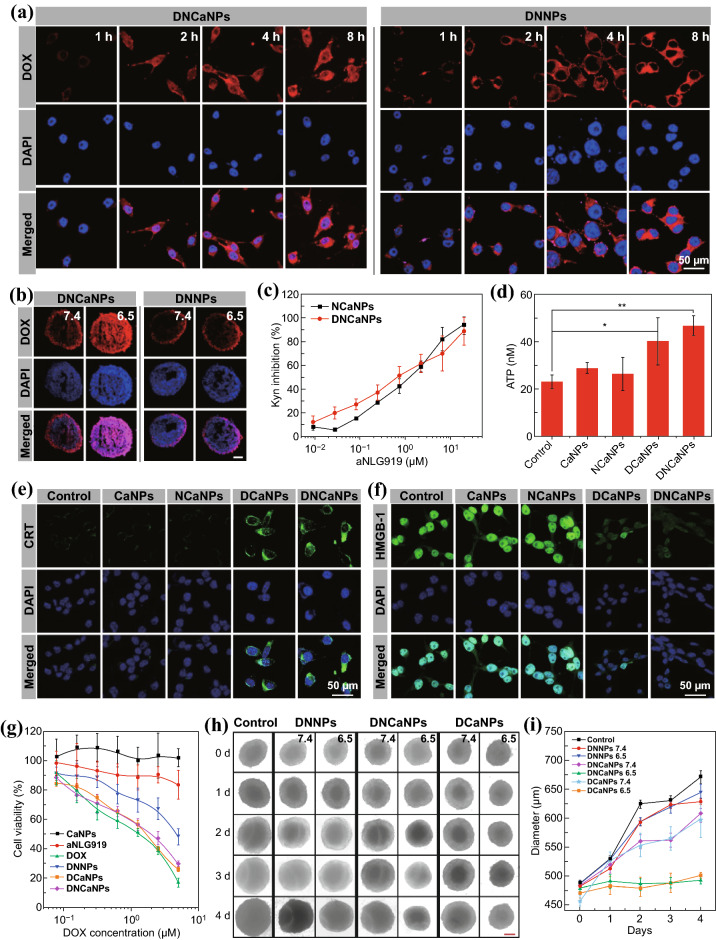
In vitro cell experiments. **a** Confocal images of CT26 cells treated by DNCaNPs and DNNPs for 1, 2, 4, and 8 h. **b** Representative confocal imaging of CT26 MCSs treated by DNCaNPs or DNNPs at pH 7.4 and 6.5 for 12 h. Scale bar was 200 μm. **c** In vitro inhibitory effects of DNCaNPs and NCaNPs on the IDO1-mediated Kyn production. **d** ATP secretion levels of CT26 cells after various treatments as indicated. **e**, **f** Confocal microscopic observation of CRT expression (**e**) and HMGB1 release (**f**) profiles of CT26 cells with various treatments as indicated. **g** Cell viability of CT26 cells treated by CaNPs, free aNLG919, free DOX, DNNPs, DCaNPs, and DNCaNPs at various concentrations for 48 h before being measured using the MTT assay. **h** Optical imaging and **i** size analysis of CT26 MCSs treated by DNNPs, DCaNPs, and DNCaNPs at pH 7.4 and 6.5. Scale bar is 200 μm

Next, the time-lapsed intracellular trafficking profiles of such DNCaNPs were carefully evaluated and compared with that of non-responsive DNNPs by using the confocal laser scanning microscopy (CLSM). It was shown that these murine CT26 cancer cells incubated with both DNCaNPs and DNNPs showed gradually increased DOX fluorescence inside the cytosol. Notably, only cells incubated with DNCaNPs showed remarkably enhanced DOX fluorescence inside the nuclei, the target of DOX, indicating the fast release of DOX from such DNCaNPs upon intracellular internalization (Fig. 2a). By incubating three-dimensional CT26 multicellular spheroids (MCSs) with DNCaNPs and DNNPs at diverse pHs for 12 h, we found that such CT26 MCSs incubated with DNCaNPs at pH 6.5 showed homogenous distribution of DOX fluorescence observed under the CLSM, in sharp contrast to these CT26 MCSs incubated with DNCaNPs at pH 7.4 and DNNPs at pH 6.5 and 7.4 (Fig. 2b). These results indicate that such DNCaNPs could enable efficient pH-responsive intratumoral penetration of therapeutics, which could be attributed to fact that free DOX molecules released from DNCaNPs at low pH would possess better diffusion ability compared to nanoparticles with much larger sizes.

### Capacities of DNCaNPs in Inhibiting IDO-1 Activity and Inducing Immunogenic Cell Death

After that, the inhibitory capacity of such DNCaNPs toward IDO1 was carefully evaluated by incubation with IDO1 high-expressed CT26 cells induced by the pretreatment with interferon-gamma (IFN-γ). As measured via the previously reported Ehrlich’s reaction, those pretreated CT26 cells upon incubation with DNCaNPs for 48 h efficiently inhibited Kyn production, which was in proportion to the concentration of aNLG919, similar to those cells treated by the incubation with NCaNPs (Fig. 2c). Moreover, it was further found that the Trp/Kyn ratios in the supernatants of these CT26 cells incubated with DNCaNPs and NCaNPs as aforementioned were also in proportion to the concentration of aNLG919 by recording the concentrations of Trp and Kyn in these supernatants using HPLC (Fig. S7). Then, the capacities of such DNCaNPs in promoting the release of the damage-associated molecular patterns (DAMPs) including adenosine triphosphate (ATP), calreticulin (CRT), and high mobility group box 1 (HMGB1), which were recognized as the markers of ICD, were carefully evaluated on CT26 cells [41, 42]. By utilizing the commercial ATP detection kit, it was shown that those CT26 cells incubated with DNCaNPs and DCaNPs (DOX = 5 μM) for 24 h showed effective release of ATP to the supernatants, while the treatment with CaNPs and NCaNPs showed an minimal effect of ATP release (Fig. 2d). As indicated by corresponding immunofluorescence staining assays, it was shown that those CT26 cells treated by DNCaNPs and DCaNPs both showed effective CRT expression on the plasma membrane and HMGB1 release from the nuclei. In contrast, the incubation with CaNPs and NCaNPs showed negligible influence on the CRT expression and HMGB1 release of those treated cells under the tested conditions (Fig. 2e, f). Based on the above observations, such DNCaNPs can effectively induce ICD of cancer cells.

Afterward, we carefully investigated the therapeutic efficacy of DNCaNPs using both monolayer CT26 cells and three-dimensional (3D) CT26 MCSs. We found that the half-maximal inhibitory concentration (IC50) of DNCaNPs against monolayer CT26 cells was 1.36 μM, which was comparable to 1.05 and 1.44 μM for free DOX and DCaNPs, respectively, but lower than 3.98 μM for non-responsive DNNPs as measured via the 3-(4,5-dimethyl-2-thiazolyl)-2,5-diphenyl-2-H-tetrazolium bromide (MTT) assay. In sharp contrast, aNLG919 and plain CaNPs showed a minimal impact on the viability of those cells (Fig. 2g). Furthermore, as monitored under the optical microscopy, it was shown that the growth of 3D CT26 MCSs treated by incubation with both DNCaNPs and DCaNPs (DOX = 10 μM) at pH 6.5 for 4 days was fully regressed compared to those treated by incubation with DNCaNPs and DCaNPs at pH 7.4 or with DNNPs at pH 7.4 and 6.5 (Fig. 2h, i). This result could mainly be attributed to that DOX in the DNCaNPs formulation has the best tumor permeability under acidic conditions owing to its faster pH-responsive release, so it could effectively inhibit the growth of 3D CT26 MCSs from the inside out.

### In Vivo Pharmacokinetics Profiles of DNCaNPs

**Fig. 3:**
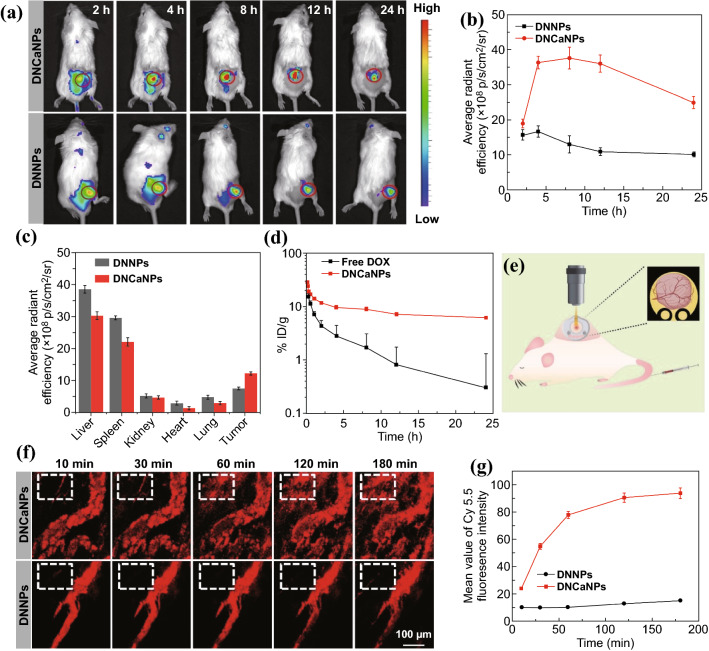
In vivo fluorescence imaging, pharmacokinetics, and tumor penetration behaviors of DNCaNPs. **a** In vivo fluorescence imaging of CT26 tumor-bearing mice intravenously injected with DiR-labeled DNCaNPs and DNNPs by recording DiR fluorescence. **b** Semiquantitative analysis of DiR fluorescence intensities of the tumor regions based on the imaging data shown in (**a**). **c** Semiquantitative analysis of DiR fluorescence intensities of the main organs as indicated and tumors based on their ex vivo imaging at 24 h post-injection of DiR-labeled DNCaNPs and DNNPs as shown in Fig. S8. **d** Blood circulation behaviors of DNCaNPs and free DOX as analyzed by measuring DOX fluorescence in the lysed blood samples. **e** Schematic illustration of the mice prepared for intravital microscopic imaging. **f** Intravital microscopic observation of time-lapsed extravasation and tumor penetration behaviors of Cy5.5 (as the mimic of DOX) inside the CT26 tumors with intravenous injection of DNCaNPs and DNNPs at designated time intervals. Scale bar is 100 μm. **g** Semiquantitative analysis of Cy5.5 fluorescence intensities of region of interest (white dashed rectangles) drawn in **f**

Afterward, we carefully evaluated the in vivo pharmacokinetics profile of such DNCaNPs. After being labeled with near-infrared (NIR) fluorescent dye of 1,1′-dioctadecyl-3,3,3′,3′-tetramethylindotricarbocyanine iodide (DiR), both DNCaNPs and DNNPs upon intravenous (i.v.) injection were shown to be able to gradually accumulate at the tumor site as visualized using the commercial in vivo optical imaging system (Fig. 3a). By semiquantitatively analyzing the DiR fluorescence intensities of tumor regions, we found that those tumors on the Balb/c mice with systemic administration of DiR-labeled DNCaNPs showed much stronger DiR fluorescence than those injected with DiR-labeled DNNPs (Fig. 3b). The superior tumor-homing capacity of DNCaNPs was further confirmed by recording the DiR fluorescence of tumors collected at 24 h post-systemic administration under the commercial optical imaging system (Figs. 3c and S8). Moreover, by recording the DOX fluorescence, the blood circulation profile of such DNCaNPs was found to follow a two-compartment model, and its first and second half-life times were determined to be 0.32 ± 0.08 and 6.85 ± 0.97 h, respectively, remarkably longer than 0.17 ± 0.05 and 1.81 ± 0.49 h for free DOX (Fig. 3d). By calculation, the area under curve (AUC) values of DNCaNPs and free DOX were determined to be 198.8 and 49.7%ID g^−1^ h, respectively.

Motived by aforementioned results that such DNCaNPs endowed highly efficient penetration of DOX into 3D CT26 MCSs at pH 6.5, we further carefully studied the penetration capacity of sulfonated cyanine 5.5 (Cy5.5) delivered by both DNCaNPs and DNNPs inside the acidic tumor microenvironment using the intravital microscopy (Fig. 3e). Here, Cy5.5 was utilized as the payload replacing DOX to avoid the interference of autofluorescence from biological tissues. It was shown that the regions aside from the tumor blood vessels within the tumor on mice with i.v. injection of Cy5.5-loaded DNCaNPs exhibited gradually increased Cy5.5 fluorescence intensity, while the Cy5.5 fluorescence was mainly around the tumor blood vessels on those mice injected with Cy5.5-loaded DNNPs (Fig. 3f, g). Taken together, those results indicate that such pH-responsive DNCaNPs is a potent nanocarrier to enable efficient tumor homing and deep intratumoral penetration of their payloads. This process is speculated as that DNCaNPs would firstly accumulate around the vessels through the defective tumor vessels and then release drug molecules in response to the acidic tumor microenvironment. Those released drug molecules with much smaller sizes compared to nanoparticles would then diffuse through tumor vessels and penetrate deeply into tumor tissues.

### In Vivo Combination Therapy on Subcutaneous CT26 Model

**Fig. 4:**
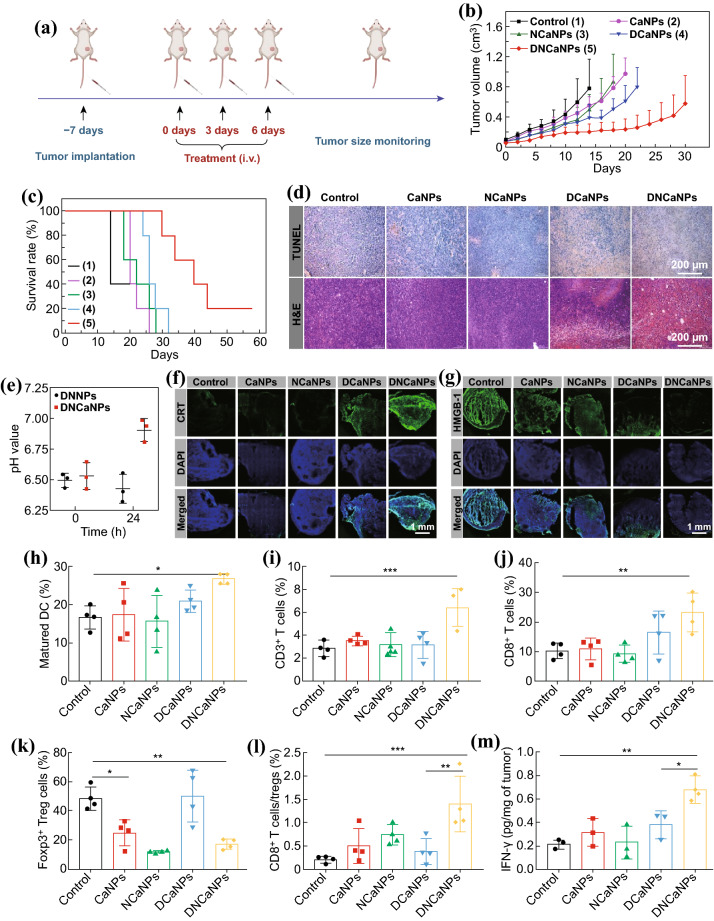
In vivo antitumor efficacy and corresponding mechanism study of DNCaNPs mediated chemo- & immunotherapy on subcutaneous CT26 tumor model. **a** A scheme illustrating the therapeutic schedule. **b**, **c** Tumor growth curves (**b**) and survival curve (**c**) of these CT26 tumor-bearing mice after being treated as indicated (*n* = 5). The mouse was set as dead when its tumor volume was over 1000 mm^3^. **d** TUNEL and H&E staining of tumor slices collected from the mice with different treatments as indicated at day 7. Scale bars are 200 μm. **e** Intratumoral pH values of tumors on mice at 0 and 24 h post-intravenous injection of plain CaNPs or PLGA-PEG nanoparticles. **f**,** g** Confocal microscopic imaging of tumor slices collected from the mice with different treatments as indicated and stained with CRT (**f**) and HMGB1 (**g**) primary antibodies. Scale bars are 1 mm. **h**, **l** DC maturation levels in draining LNs (**h**), intratumoral infiltration frequencies of CD3^+^ T lymphocytes (**i**), CD3^+^CD8^+^ T cells (**j**), and CD3^+^CD4^+^Foxp3^+^ Tregs (**k**), and the ratios of CD3^+^CD8^+^ T cells to corresponding CD3^+^CD4^+^Foxp3^+^ Tregs (**l**) in the CT26 tumors with various treatments as indicated (*n* = 4). **m** Intratumoral IFN-γ secretion in the CT26 tumors with various treatments as indicated (*n* = 3). P values was calculated by using one-way analysis of variance (ANOVA), *p < 0.05, **p < 0.01, ***p < 0.001

We then carefully evaluated the in vivo antitumor efficacy of DNCaNPs. When their tumor volumes reached ~ 100 cm^3^, mice bearing CT26 mouse colon cancer tumors were randomly divided into five groups and received corresponding treatments as follows: group 1, saline injection; group 2, CaNPs injection; group 3, NCaNPs injection; group 4, DCaNPs injection, and group 5, DNCaNPs injection. All mice received three repeated i.v. injections as indicated at day 0, 3, and 6. For corresponding groups, the injection dose of DOX was 5 mg kg^−1^, while that of aNLG was 25 mg kg^−1^ for each injection (Fig. 4a). It was found that such DNCaNPs most effectively suppress the growth of subcutaneous CT26 tumors with 1 of 5 mice cured, the median survival time of these DNCaNPs treated mice was 40 days (those mice were set as dead when their tumor volume was larger than 1000 mm^3^, Figs. 4b, c and S9). In contrast, the median survival time of those mice treated by injection of CaNPs, NCaNPs, or DCaNPs were 20, 22, and 26 days, which were only slightly longer than 14 days for mice in the control group.

Moreover, the early therapeutic efficacy of such DNCaNPs was also evaluated via the hematoxylin–eosin (H&E) staining and TdT-mediated dUTP nick-end labeling (TUNEL) staining. It was uncovered that the tumor slice of the mice collected at 1 day after the triple injections (day 7) of DNCaNPs and stained via the TUNEL staining showed effective cell apoptosis, which was much severe than those after other treatments (Fig. 4d). In addition, H&E-stained tumor slices indicated the same trend to that of TUNEL staining, showing most severe histological damages in tumors collected from mice treated by DNCaNPs.

Furthermore, we carefully explored the effects of such DNCaNP treatment on the TME to unfold their highly effective therapeutic efficacy. We speculated that CaCO_3_ nanoparticles could react with H^+^ to neutralize the acidic tumor pH, which is a key feature of TME that leads to immunosuppression [43–45]. In fact, the pH values within tumors on mice with either CaNPs treatment or PLGA-PEG nanoparticles treatment were monitored by an invasive pH microelectrode to confirm the neutralization of acidic tumor microenvironment by CaCO_3_. Notably, i.v. injection of CaNPs could indeed neutralize the acidic tumor microenvironment at 24 h post-injection, while the injection of PLGA-PEG nanoparticles without CaCO_3_ showed little effect to the pH within tumor (Fig. 4e).

### Analysis of Antitumor Mechanism

The capacities of DNCaNPs and other formulations in causing ICD of cancer cells were carefully investigated by evaluating the intratumoral CRT expression and HMGB1 release of these treated tumors collected at 1 day (Day 7) after various treatments. Via the immunofluorescence staining, we found that the treatments of both DNCaNPs and DCaNPs remarkably promoted the expression of CRT and the release of HMGB1, while the other treatments showed limited influence on the CRT expression and HMGB1 release (Fig. 4f, g). Collectively, these results indicated that the treatment of DNCaNPs injections could elicit effective cell death in the ICD manner. Then, the potency of such DNCaNPs mediated ICD of cancer cells in priming antitumor immunity was carefully evaluated by detecting the maturation levels of dendritic cells (DCs), the initial step of antitumor immunity. We found that the maturation status of these DCs isolated from the lymph nodes close to the tumors of these DNCaNP-treated mice was significantly elevated, while the other treatments showed minimal effects on the DC maturation as analyzed at day 7 via the flow cytometry (Figs. 4h and S10a). These results demonstrate that such DNCaNPs are able to induce an effective ICD of cancer cells and thus initiate an effective DC maturation.

Then, by analyzing the single-cell suspension of the tumors collected from mice with different treatments via the flow cytometry, it was shown that DNCaNPs injection could significantly promote the intratumoral frequencies of CD3^+^ T lymphocytes as well as CD3^+^CD8^+^ T lymphocytes, while the other treatments exhibited limited influences on the intratumoral frequencies of those T lymphocytes (Figs. 4i, j and S10b). In addition, we found that DNCaNPs injection could remarkably restrict the intratumoral frequency of immunosuppressive Tregs (CD3^+^CD4^+^FoxP3^+^), and the injection of plain CaNPs could even also significantly reduce the intratumoral frequency of Tregs (Figs. 4k and S10c). The latter should be ascribed to the fact that high accumulation of such CaNPs could efficiently neutralize the acidic TME, thereby reducing the intratumoral frequency of Tregs according to the previous study [46]. As a result, the ratio of CD3^+^CD8^+^ T cells to Tregs inside the tumors grown on the mice treated by DNCaNPs administration was 6.4- and 3.6-folds in comparison with those of the mice treated by saline and DcaNPs injections, respectively (Fig. 4l). Moreover, we found that the secretion level of cytotoxic IFN-γ inside the tumors grown on the DNCaNP treated mice was quantified to 3.2-folds compared to that of control groups, remarkably higher than 1.8-folds for the mice with DCaNPs injection (Fig. 4m). Taken together, those results demonstrated that such DNCaNPs could synergistically prime effective antitumor immune responses as evidenced by the increased DC maturation, elevated frequency of tumor-infiltrating CD3^+^CD8^+^ T lymphocytes and expression of cytotoxic IFN-γ inside tumors, owing to the DOX-induced immunogenic cancer cell death, the neutralization of acidic TME by CaCO_3_, as well as NLG-919-mediated reverse of immunosuppressive TME.

### In Vivo Combination Therapy on Orthotopic 4T1 Model

**Fig. 5:**
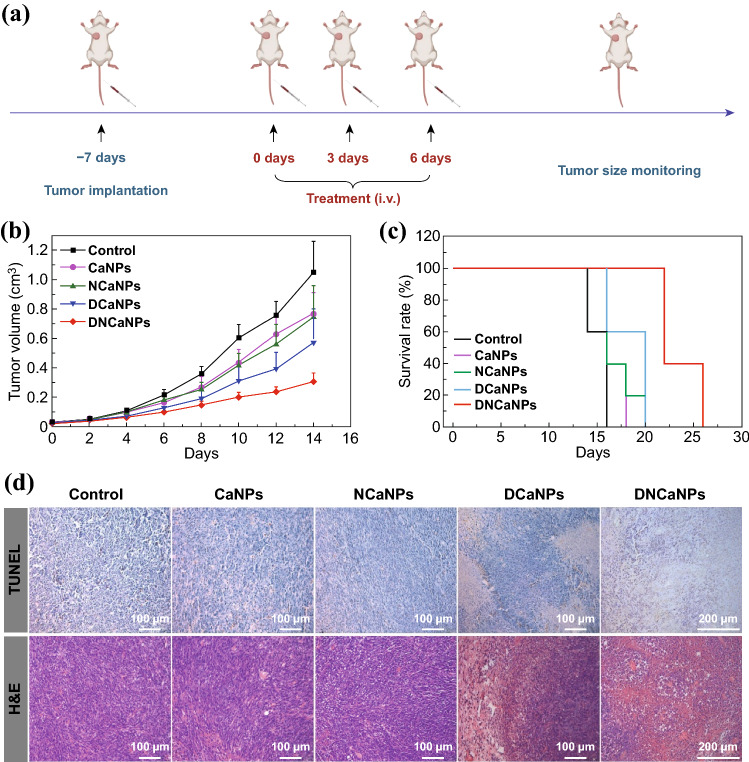
In vivo antitumor efficacy of the DNCaNP-mediated chemo- & immunotherapy on orthotopic 4T1 tumor models. **a** A scheme illustrating the therapeutic schedule. **b, c** Tumor growth curve (**b**) and corresponding survival curves (**c**) of these 4T1 tumors on the mice with various treatments as indicated (*n* = 5). The mouse was set as dead when its tumor volume was over 1000 mm^3^. **d** TUNEL and H&E staining of tumor slices collected from the mice with various treatments as indicted at day 7. Scale bar is 200 μm

Inspired by the high efficacy of such DNCaNPs in priming antitumor immune responses on the subcutaneous CT26 colorectal tumor model, we further investigated the therapeutic potency of such DNCaNPs on the poorly immunogenic orthotopic 4T1 tumor model of triple-negative breast cancer. As being treated by following the therapeutic regimen for those subcutaneous CT26 tumors (Fig. 5a), these orthotopic 4T1 tumors on mice with triple injections of DNCaNPs were the most effectively regressed, and their median survival time was calculated to be 24 days (Figs. 5b, c and S11), while the other treatment showed limited inhibitory effect on those orthotopic 4T1 tumors. Additionally, the superior therapeutic efficacy of such DNCaNPs was further confirmed via the H&E and TUNEL staining of the tumor slices of these tumor-bearing Balb/C mice post-distinct treatments as aforementioned (Fig. 5d). Obvious cell apoptosis and histological damages were observed on these tumor slices of mice post-DNCaNPs treatment via the TUNEL and H&E staining, respectively, indicating that such DNCaNPs would be highly effective for cancer therapy.

## Conclusions

**Scheme 1:**
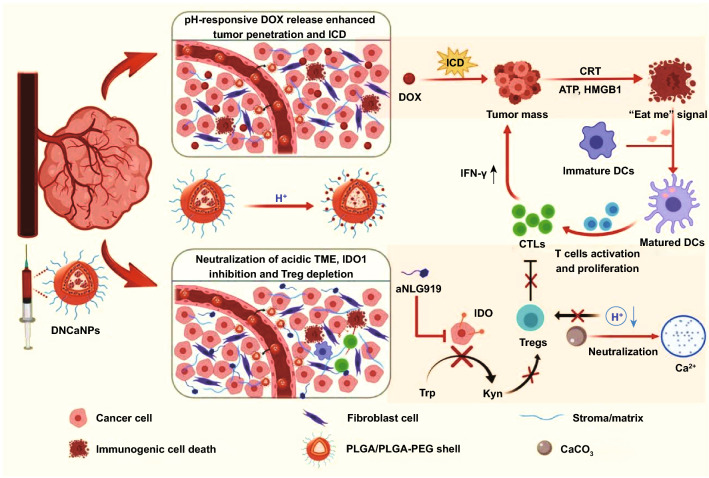
Schematic illustration of the preparation of tumor extracellular pH-responsive immune-modulatable DNCaNPs for effective cancer chemo- & immunotherapy. Upon intravenous injection, such DNCaNPs would passively accumulate at tumor site and rapidly release the loaded DOX in response to the acidic TME, thereby enabling efficient tumor penetration of DOX. Such DOX would induce effective ICD of cancer cells featured in enhanced release of DAMPs of ATP, CRT and HMGB1 as the “eat me” signals to host’s immune system, thus priming the host’s specific antitumor immunity by promoting the maturation of DCs, intratumoral infiltration of effector T cells, and secretion of cytotoxic IFN-γ. In the meantime, tumor-accumulated CaCO_3_ component would neutralize the acidic TME by reacting with H^+^ within tumors, and the released aNLG919 would potently inhibit the IDO1-mediated production of immunosuppressive Kyn, thereby synergistically restricting the intratumoral frequency of immunosuppressive Tregs. As a result, such DNCaNPs would prime profound antitumor immune response and efficiently inhibit the tumor growth via the chemo- & immunotherapy

In this work, we developed a concise CaCO_3_-assisted double emulsion method to prepare pH-responsive immune-modulating nanoparticles to enable highly efficient chemo-immunotherapy. The introducing of CaCO_3_ could remarkably elevate the loading efficiencies of therapeutic agents compared to conventional PLGA-based delivery systems. Meanwhile, owing to the reaction of CaCO_3_ with H^+^, those drug-loaded CaNPs with pH-responsive dissociate behaviors not only allow deep intratumoral penetration of therapeutic agents, but also enable effective neutralization of acidic tumor pH to favor the reverse of immunosuppressive TME (Scheme 1).

With co-loading of chemotherapeutic agent DOX as the ICD inducer and aNLG919 as an IDO1 inhibitor, DNCaNPs after i.v. injection could induce effective immunogenic tumor cell death, thus promoting DCs maturation, CD3^+^CD8^+^ T lymphocyte infiltration, and cytotoxic IFN-γ secretion, as well as in meanwhile significantly restricting intratumoral frequency of immunosuppressive Tregs. Such reverse in the immunosuppressive TME features could probably be ascribed to NLG919-mediated IDO1 inhibition and CaCO_3_-mediated neutralization of acidic TME. As a result, DNCaNPs are able to elicit potent antitumor efficacy on these subcutaneous CT26 mouse colon tumors and orthotopic 4T1 triple-negative mouse breast tumors, the latter of which is known to be a poorly immunogenic tumor model resistant to diverse immune stimulation-based treatments [47–49].

In summary, this work presents a CaCO_3_-containing nanomedicine formulation that could allow effective modulation of immunosuppressive TME to achieve enhanced chemo-immunotherapy of cancer. Considering that both CaCO_3_ and PLGA-PEG are well known as well biocompatible components, our DNCaNPs would be highly safe and promising for future clinical translation.

## Electronic supplementary material

Below is the link to the electronic supplementary material.

Supplementary file1 (PDF 1114 kb)
