# Supplementary material for: CaCO3-Assisted Preparation of pH-Responsive Immune-Modulating Nanoparticles for Augmented Chemo-Immunotherapy
Source: Nanomicro Lett. 2020 Nov 22;13:29. doi: 10.1007/s40820-020-00549-4 (PMC8187673; doi:10.1007/s40820-020-00549-4)
Supplement: Supplementary file 1 — Supplementary file1 (PDF 1114 kb) [file 40820_2020_549_MOESM1_ESM.pdf]

Supporting Information for

$CaCO_3$-Assistant Preparation of pH-Responsive Immune-Modulating Nanoparticles for Augmented Chemo-Immunotherapy

Yujie Zhu[1], Zhijuan Yang[1], Ziliang Dong[1], Yimou Gong[1], Yu Hao[1], Longlong Tian[1], Xianzhu Yang[2], zhuang Liu[1,] *, Liangzhu Feng[1,] *

[1] Jiangsu Key Laboratory for Carbon-Based Functional Materials and Devices, Institute of Functional Nano and Soft Materials (FUNSOM), Soochow University, Suzhou, Jiangsu 215123, People's Republic of China

[2] Institutes for Life Sciences, School of Medicine, South China University of Technology, Guangzhou, Guangdong 510006, People's Republic of China.

*Corresponding authors. E-mail: lzfeng@suda.edu.cn (Liangzhu Feng), zliu@suda.edu.cn (zhuang Liu)

Supplementary Table and Figures

Table S1 Mean diameters and polydispersity indexs of DNCaNPs and DNNPs

	Mean diameter (Number %)	Polydispersity index
DNCaNPs	178.5 ± 5.9nm	0.061 ± 0.03
DNNPs	146.9 ± 8.7nm	0.100 ± 0.047

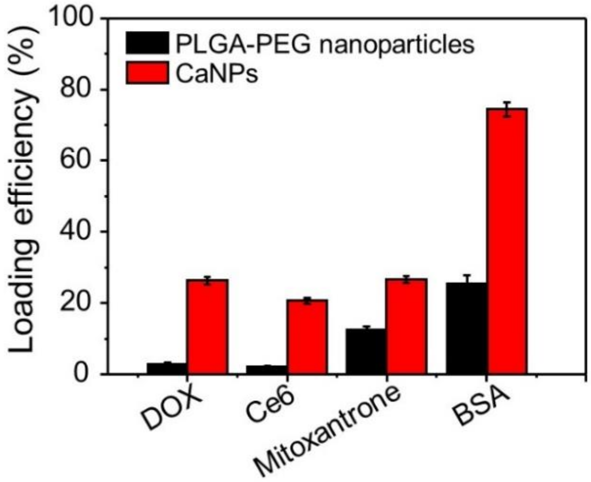

Fig. S1 Comparison of the loading efficiencies of CaNPs prepared via our developed $CaCO_3$ assisted double emulsion method and counterpart PLGA-PEG nanoparticles prepared via the classical double emulsion method towards DOX, Ce6, mitoxantrone and BSA and at a fixed payload to PLGA/PLGA-PEG mass ratio of 1:15. The amounts of DOX, Ce6 and mitoxantrone were quantified via the spectrometry, while the amounts of BSA was quantified using the BCA kit.

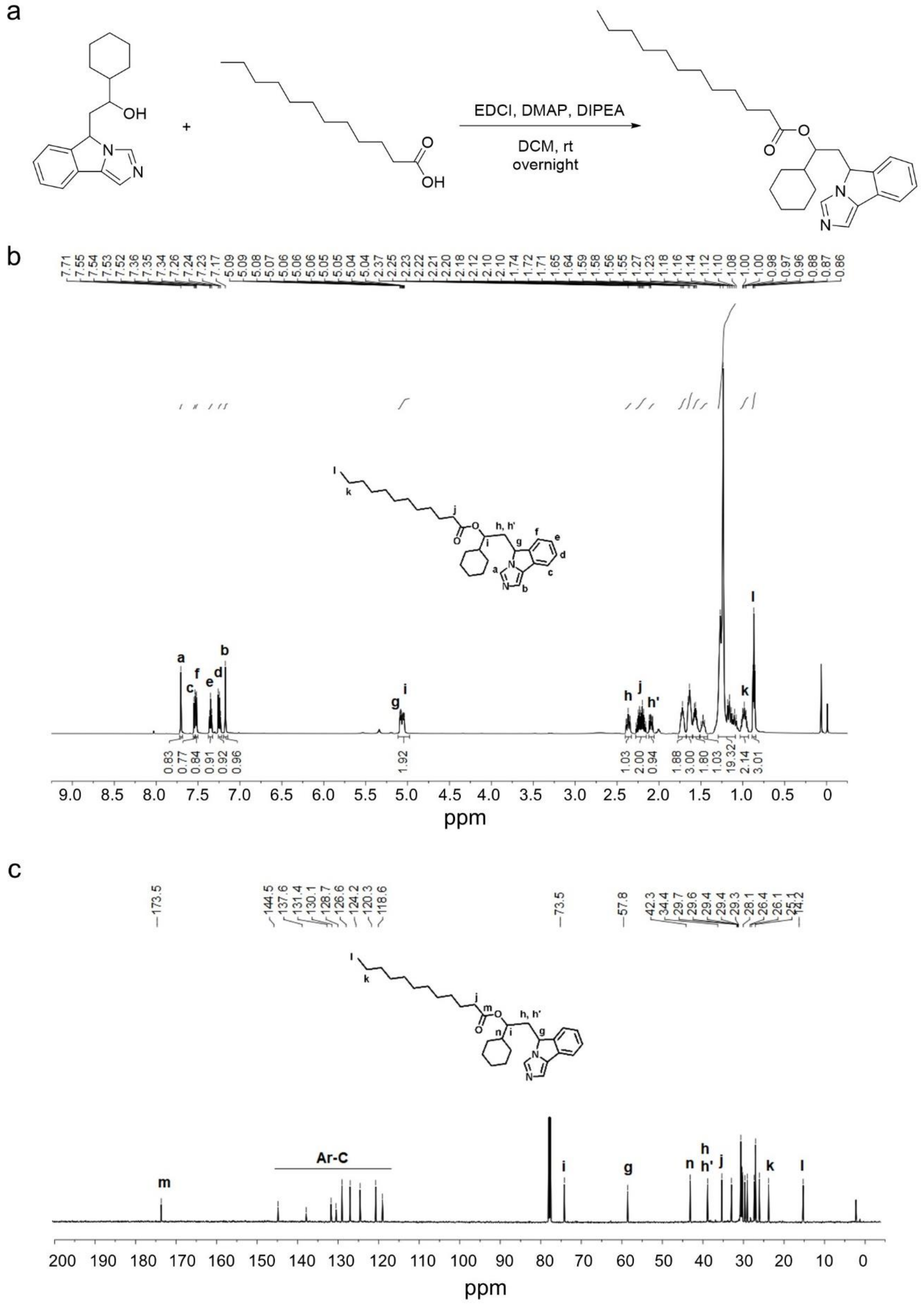

Fig. S2 Synthesis and characterization of aNLG919. (**a**) A scheme illustrating the synthetic route of aNLG919. (**b**) ^{1}H-NMR spectrum and (**c**) ^{13}C-NMR spectrum of such aNLG919

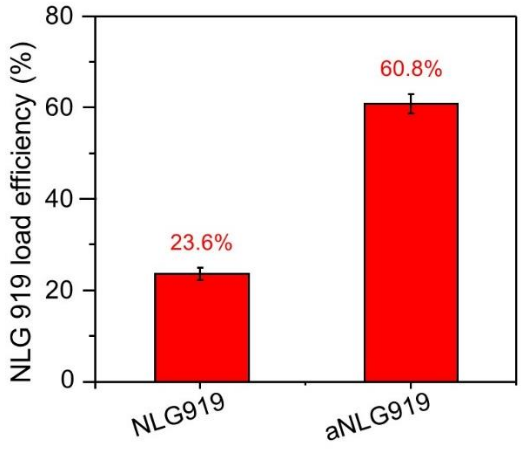

Fig. S3 Loading efficiencies of NLG919 and aNLG919 by CaNPs prepared at a mass feeding ratio of NLG919 or aNLG919 to PLGA/PLGA-PEG = 2:15

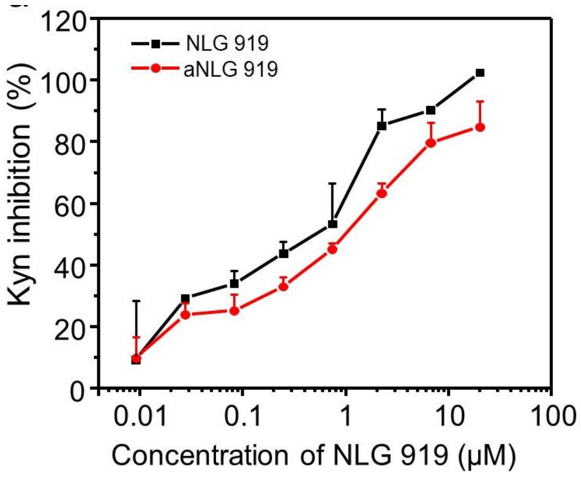

Fig. S4 In vitro inhibitory effects of NLG919 and as-synthesized aNLG919 on IDO1 mediated production of Kyn

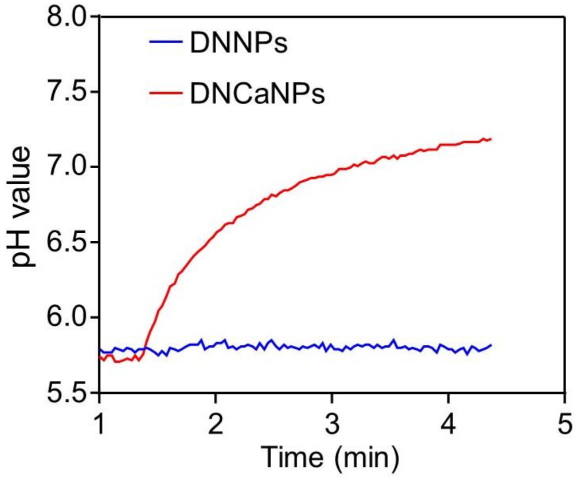

Fig. S5 In vitro acidic neutralization of DNCaNPs

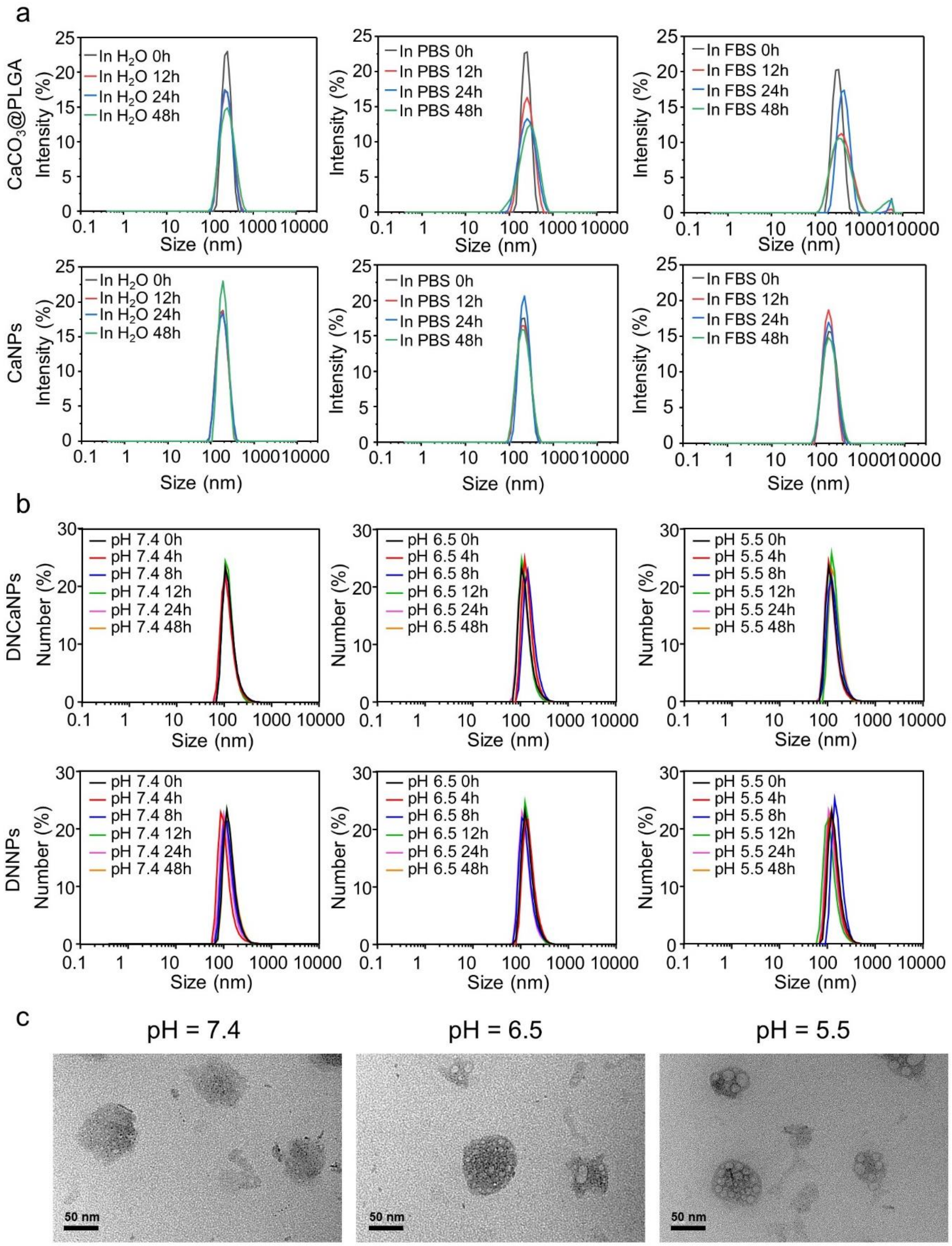

Fig. S6 Physiological stability evaluation. (**a**) DLS size distribution profiles of CaNPs and its counterpart $CaCO_3$@PLGA nanoparticles without PLGA-PEG incubated in pure water, PBS and FBS solutions for different time intervals as indicated. (**b**) DLS size distribution profiles of DNCaNPs and DNNPs incubated at pH 5.5, 6.5, and 7.4 for different time intervals. (**c**) Representative TEM images of DNCaNPs incubated at pH 5.5, 6.5, and 7.4 for 24 h

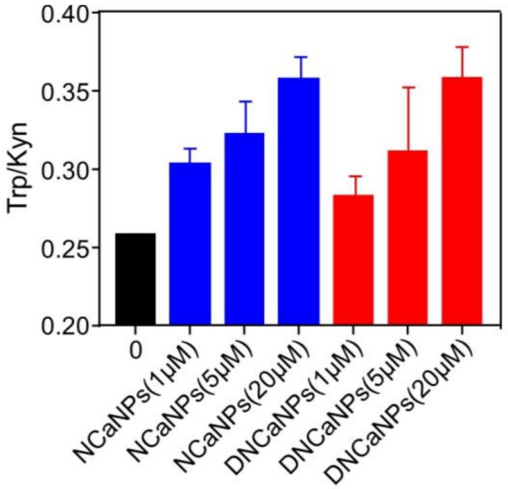

Fig. S7 In vitro effects of NcaNPs and DNCaNPs on the ratio of Trp to Kyn

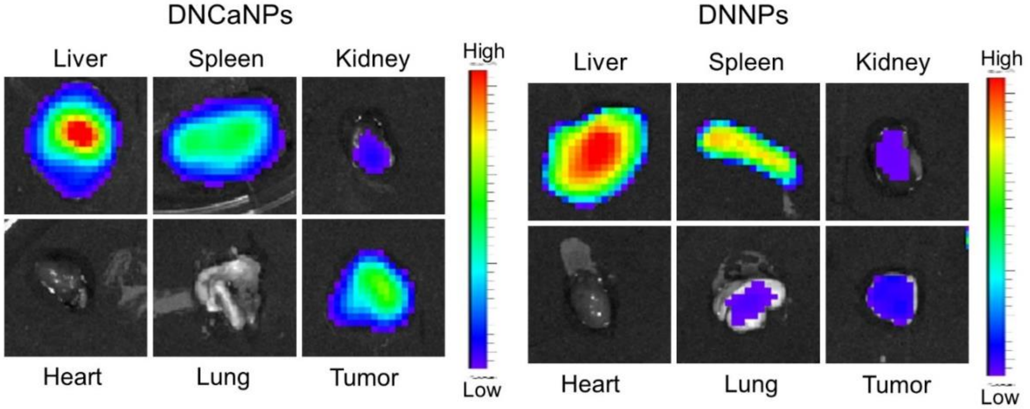

Fig. S8 Ex vivo fluorescence imaging of the organs as indicated collected from the mice with intravenous injection of DiR labeled DNCaNPs or DNNPs at 24 h p.i.

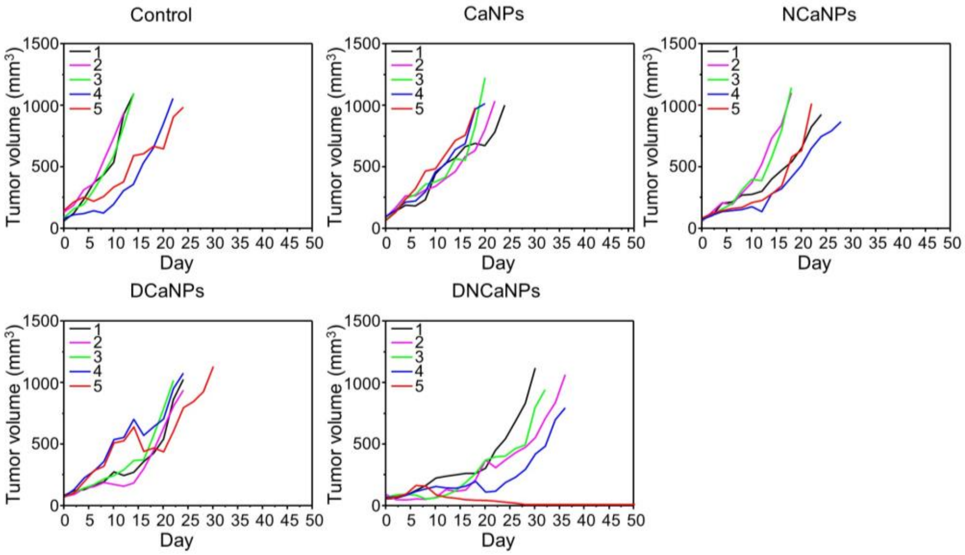

Fig. S9 Individual tumor growth curves of these CT26 tumor bearing mice after being treated as indicated (n = 5). The mouse was set as dead when its tumor volume was over 1000 mm^3

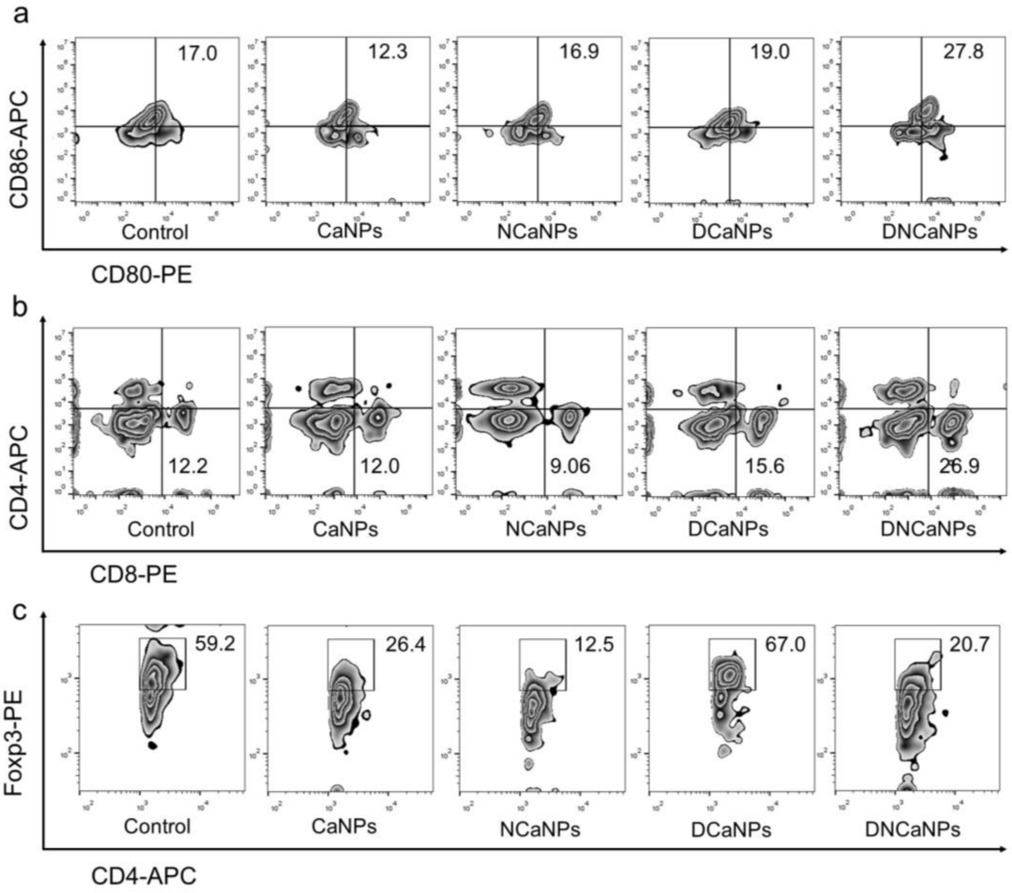

Fig. S10 Flow cytometric analysis of maturation levels of DC cells in the draining lymph nodes (**a**), intratumoral infiltration of $CD3^{+}CD8^{+}$ T cells (**b**) and Tregs (**c**) on the CT26 tumor bearing mice post various treatments as indicated (n = 5)

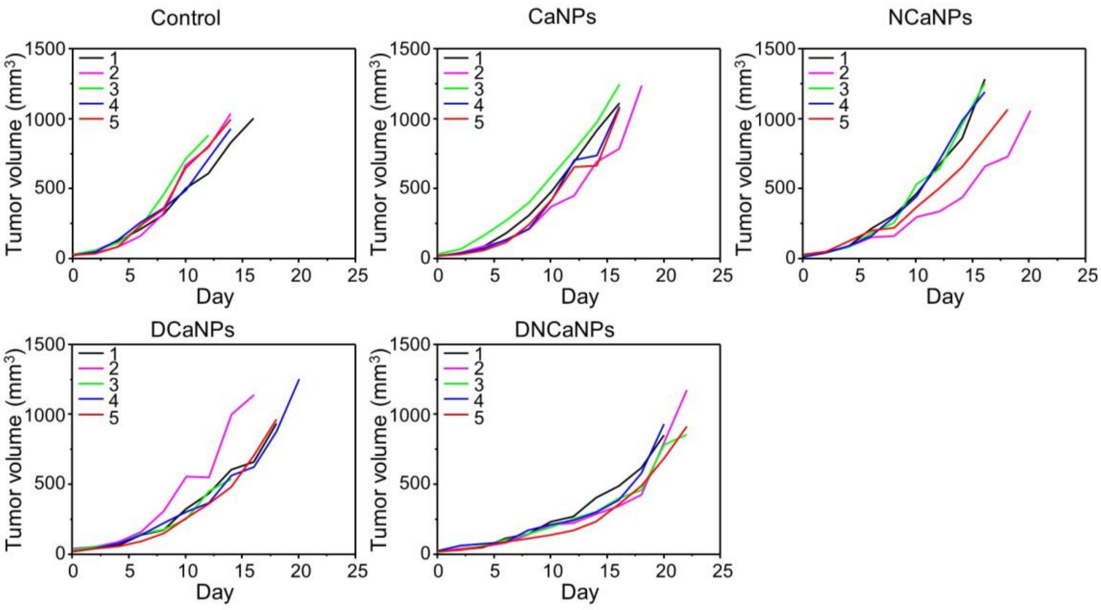

Fig. S11 Individual tumor growth curves of these 4T1 tumor bearing mice after being treated as indicated (n = 5). The mouse was set as dead when its tumor volume was over 1000 mm^3
